# Total endovascular repair of a malpositioneted frozen elephant trunk with Thoraflex hybrid prosthesis: A case report

**DOI:** 10.34172/jcvtr.2022.24

**Published:** 2022-06-30

**Authors:** Francesco Buia, Luca Di Marco, Davide Pacini, Luigi Lovato

**Affiliations:** ^1^Cardio-Thoracic-Vascular Radiology Unit, Cardio-Thoracic-Vascular Department, S.Orsola Hospital, University of Bologna, Italy; ^2^Cardiac Surgery Unit, Cardio-Thoracic-Vascular Department, S.Orsola Hospital, University of Bologna, Italy

**Keywords:** Aortic Dissection, Endovascular Repair, Frozen Elephant Trunk

## Abstract

We report a case of a 56-year-old male who underwent Frozen Elephant Trunk procedure for residual type A chronic aortic dissection, complicated by the release of the distal endovascular portion of the hybrid prosthesis in the false lumen. This complication was successfully treated with a totally endovascular approach.

## Introduction

 Over the last decade, the Frozen Elephant Trunk (FET) technique allowed to treat complex diseases of the aortic arch and thoracic aorta, with the use of a hybrid prosthesis with a proximal conventional surgical vascular Dacron-graft and a distal endovascular nitinol-stent graft. The most feared complications are stroke and spinal cord injury and, when is needed, second stage procedures are more frequently endovascular.^1^ We report the case of a patient with residual type A chronic aortic dissection treated with the FET technique complicated by the release of the distal endovascular stent graft in the false lumen, successfully treated with a second stage endovascular repair. The aim of this case is to show a minimally invasive option to solve this type of possible complication.

## Case Presentation

**Figure 1:**
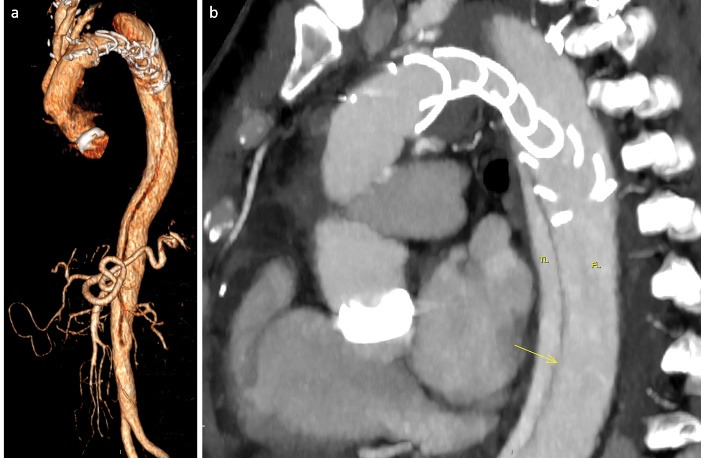
CT scan control of FET shows the Thoraflex endoprosthesis in the false lumen (a) and the intimal tear (arrow, b) in the mid tract of descending thoracic aorta (TL = true lumen; FL = false lumen).

**Figure 2:**
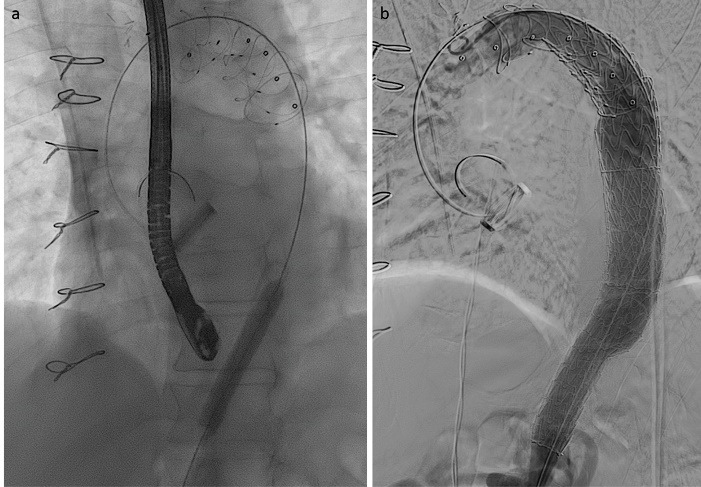
Intraoperative cannulation and dilation of the thoracic intimal tear (a) and angioscopy control after deployment of the stent graft (b).

**Figure 3:**
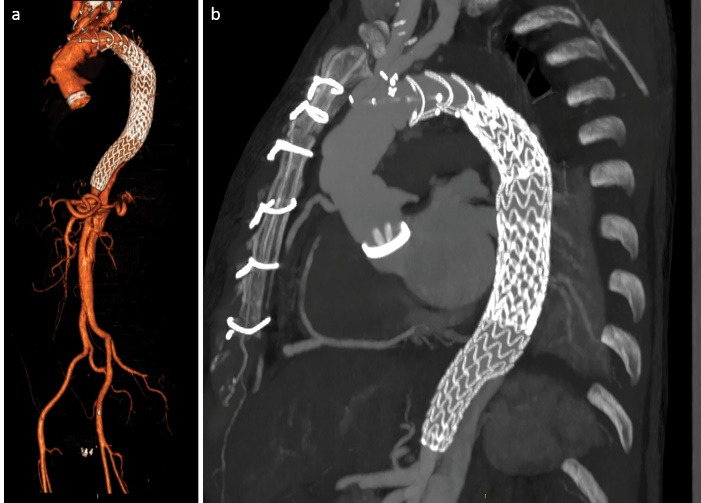
CT scan control at discharge (a) and 2 years follow-up (b) showing complete exclusion and thrombosis of the thoracic false lumen.

 A 56-year-old male, previously operated with mechanical Bentall procedure and right coronary artery bypass grafting for acute type A aortic dissection, was admitted to our hospital with the diagnosis of residual type A chronic aortic dissection to undergo FET procedure. Total arch replacement with FET was performed in operative room (OR) using a 28/30 mm Thoraflex hybrid prosthesis (Vascutek, Inchinnan, UK). Briefly, after circulatory arrest and cerebral protection with selective bilateral antegrade cerebral perfusion at a target nasopharyngeal temperature of 25 °C, the hybrid prosthesis was deployed into the descending thoracic aorta. Distal anastomosis was performed at Ishimaru zone 2 of the aortic arch. Systemic perfusion was re-established through one of the side-branch of the hybrid prosthesis and then, the supra-aortic vessels re-implantation and the proximal anastomosis were completed. The post-operative period was uneventful although the pre-discharged computed tomography angiography (CT) scan control showed the incorrect deployment of the distal endovascular portion of the Thoraflex in the false lumen (Figure 1a). The residual flap length was about 25cm, extending from the middle tract of thoracic aorta to the sub-renal tract; celiac trunk, superior mesenteric artery and right renal artery originated from true lumen, left renal artery from false lumen. Then, the aortic team decided to perform an angiography to evaluate the feasibility to access the false lumen through an intimal tear visualized in the pre-discharged CT examination in the middle portion of the thoracic aorta (Figure 1b), in order to evaluate the possibility to create a communication between the stent graft portion of the Thoraflex in the false lumen and the true lumen. After the angiographic confirmation of the feasibility of the procedure, the patient was then scheduled to perform a thoracic endovascular aortic repair (TEVAR). In a hybrid operative room, with the support of intraoperative transesophageal echo-guidance, the access to the false lumen was through the cannulation of the intimal tear located in the middle tract of the thoracic aorta. Then, the cannulated tear was dilated with the use of balloon catheter (Medtronic Evercross Pta Baloon Catheter, 6x60mm and 8x60mm, Minneapolis, MN) (Figure 2a). After positioning of an ultra-stiff guide wire through the dilated tear, two stent grafts were released proximally (Gore Tag Thoracic Stent Graft 28 mm x 28 mm x 150 mm, Usa AZ) in the portion of the Thoraflex deployed into the false lumen and distally (Gore Tag Thoracic Stent Graft 31 mm x 31 mm x 150 mm, Usa AZ) in the true lumen of thoracic aorta above the celiac trunk (Figure 2b). The intraoperative angiography showed the successful outcome of the procedure (Figure 2b); the postoperative period was uneventful and the patient was discharged in seventh post-procedural day. The pre-discharged CT scan control confirmed the complete exclusion and thrombosis of the false lumen in the thoracic aorta (Figure 3a). The CT scan performed at two years follow-up confirmed the good result of the procedure (Figure 3b).

## Discussion

 Complex lesions of the arch and of the descending thoracic aorta represent a challenge for cardiovascular surgeons. From 2003 the FET technique has gained extremely popularity for the treatment of complex diseases of the aortic arch and descending thoracic aorta, most of the time in single-step procedure.^1^ When necessary, in case of distal aneurysmal dilatation, endoleaks or distal stent graft induced new entry,^2^ the second stage procedures are more frequently endovascular. Fuji et al. and Kawashima et al. already published incorrect deployment of the FET into the false lumen.^3,4^. In the case reported by Fuji,^3^ despite extensive enterectomy and abdominal aorta fenestration, the patient died of multiorgan failure. Kawashima et al.^4^ performed a successful TEVAR through a fenestration made into the intimal ﬂap, because wiring through the existing lumens was not technically possible; in this case, the patient was discharged on postoperative day 67 (after acute renal failure and gastrointestinal bleeding without further complications, and open abdomen bifurcated grafting for an abdominal aortic aneurysm). Furthermore, in both of the cases, the endovascular repair of this rare complication was performed in emergency. Tamai et al.^5^ also reported a case of a FET malposition successfully diagnosed intraoperatively and resolved with additional FET deployment into the true lumen. Moreover, Takagi et al. ^6^ recently described a case of FET malposition into the false lumen confirmed intraoperatively by intravascular ultrasound (IVUS); in this case percutaneous fenestration and subsequent endograft deployment from the FET to the true lumen was performed. In our case, a successful total endovascular repair with election criteria and planning the procedure and without necessity of aortic fenestration was possible, due to the clinical stability of the patient and the anatomic configuration of the thoraco-abdominal aorta

## Conclusion

 This rare case confirms how some technical aspects, such as the intraoperative use of angioscopy to view inside the descending thoracic aorta before and immediately after the release of the hybrid prosthesis, and the placement, under transesophageal control and after systemic heparinization, of a guide-wire in the thoracic aorta through the femoral artery, are crucial to the success of the FET above all in aortic dissection in order to correctly position the stent-graft portion of the hybrid prosthesis and to be sure to release the prosthesis into the true lumen,.^7^ In addition to these solutions, the use of IVUS could also play an important role to prevent this fearful complication. Another fundamental aspect emerging from this case is the need of a meticulous evaluation of the pre-discharged CT scan, in order to early diagnosing complications and to choose and simulate the appropriate solution. In conclusion, in case of FET with an uncorrect release of the distal stent graft in the false lumen, when the aortic anatomy and the relationship between true and false lumen allows it, TEVAR could represent a very useful option to successfully treat this complication.

## Funding

 No sources of support for the research and no grant-funding agency for me and for all of the Authors.

## Ethical approval

 Our institutional review board approved this study; requirement for informed consent was waived because of the retrospective nature of the patients’ data.

## Competing interest

 No conflicts of interest are present for me and for all of the Authors.

## References

[1] Shrestha M, Martens A, Kaufeld T, Beckmann E, Bertele S, Krueger H (2017). Single-centre experience with the frozen elephant trunk technique in 251 patients over 15 years. Eur J Cardiothorac Surg.

[2] Wang R, Weng G, Huang L, Chen Z, Huang X, Xue Y (2018). Covered stent graft for distal stent graft-induced new entry after frozen elephant trunk operation for aortic dissection. Ann Cardiovasc Thorac Surg.

[3] Fujii M, Watanabe H, Otsu M, Sugahara Y (2019). Incorrect frozen elephant trunk deployment into the false lumen of a patient with complicated type B acute dissection. Eur J Cardiothorac Surg.

[4] Kawashima M, Nomura Y, Matsumori M, Murakami H (2020). Bail-out thoracic endovascular aortic repair for incorrect deployment of frozen elephant trunk into the false lumen. Interact Cardiovasc Thorac Surg.

[5] Tamai K, Hori D, Yuri K, Yamaguchi A (2020). Additional frozen elephant trunk as a bailout for a misdeployed frozen elephant trunk in the false lumen in a patient with acute aortic dissection. Eur J Cardiothorac Surg.

[6] Takagi D, Wada T, Igarashi W, Kadohama T, Kiryu K, Arai T (2021). Endovascular rescue for malpositioned frozen elephant trunk into the false lumen. J Card Surg.

[7] Di Marco L, Murana G, Fiorentino M, Amodio C, Mariani C, Leone A (2019). The frozen elephant trunk surgery: a systematic review analysis. Indian J Thorac Cardiovasc Surg.

